# NPS MedicineWise: 20 years of change

**DOI:** 10.1186/s40545-018-0145-y

**Published:** 2018-08-01

**Authors:** Lynn Maria Weekes, Suzanne Blogg, Sharene Jackson, Kerren Hosking

**Affiliations:** NPS MedicineWise, 418A Elizabeth St, Surry Hills, Australia

**Keywords:** Quality use of medicines, Health technology assessment, Rational use of medicines, National medicines policy, Health technology education

## Abstract

The cost and potential harms of medicines and other health technologies are issues of concern for governments and third party payers of health care. Various means have been demonstrated to promote appropriate evidence-based use of these technologies as a way to reduce waste and unintended variation. Since 1998, Australia has had a national organisation responsible for large scale programs to address safe, effective and cost effective use of health technologies. This article reviews 20 years of experience for NPS MedicineWise (NPS).

NPS provides evidence-based information to health professionals and consumers using interventions that have been shown to be effective. A mix of academic detailing, audit and feedback and interactive learning is built into national programs designed to improve the use of medicines and medical tests. The target audiences have typically been general practitioners, pharmacists and nurses in primary care. Consumer programs, including mass media campaigns have supported the work with health professionals.

NPS receives most of its income from the Australian Government and in return it is required to show saving for the Pharmaceutical Benefits Scheme and the Medical Benefits Schedule. Since 1998, total savings of AUD 1096.62 million have been demonstrated. In addition, changes in knowledge and attitudes, changes in prescribing and test ordering behaviours and improvements in health outcomes have been shown through annual evaluations.

## Background

Optimising use of medicines and medical tests is of interest for health systems as costs rise and there are concerns about safety and quality. NPS MedicineWise [NPS] has been working since 1998 for safe, effective and cost-effective use of medicines. NPS has been an experiment in creating change, nationally-scaled, at ‘arms-length’ from government and underpinned by quality use of medicines (QUM) principles.

In the 1980s, the World Health Organisation promoted the *Rational Use of Drugs* and called for the establishment of National Medicinal Drug Policies [1]. The Consumers’ Health Forum of Australia (CHF) published *Towards a National Medicinal Drug Policy in Australia* which identified three critical components: quality of the product; quality use and equitable access to medicines [2]. This leadership from health consumers has been integral to the QUM movement in Australia.

A watershed meeting in 1991, co-hosted by CHF and the Australasian Society for Clinical Pharmacologists and Toxicologists [ASCEPT] on rational prescribing, called for a national drug policy, national therapeutic guidelines, a prescribing curriculum for medical students, more clinical pharmacologists and appropriate medicines information for patients [3].

The meeting galvanised activity and the Pharmaceutical Health and Rational use of Medicines (PHARM) Committee was established. It funded some $10 million of projects that improved the use of medicines [4] but when projects ended there was no way to sustain interventions [5].

Concurrently the Australian Pharmaceutical Advisory Council (APAC) was established to advise the Minister for Health on the National Medicinal Drug Policy. This was a representative council [6] and it developed guidance on: medication management in aged care [7]; Consumer Medicines Information (CMI) [8, 9]; privacy and health data [10]; and the continuum of care [11].

Based on PHARM’s recommendations for a national centre to coordinate QUM activity, NPS came into being. This paper outlines 20 years of activity and lessons learnt during that journey.

### Establishment

The 1997 Australian Government announced $22 million to establish the National Prescribing Service which aimed to improve health outcomes by supporting quality prescribing. Stakeholders identified a need for coordinated and independent prescribing support services and for this reason NPS was incorporated as an independent company, not a part of government [12].

An advisory group oversaw establishment and through a national consultation produced 13 recommendations (Table 1). The consultation emphasised the importance of establishing credibility, generating early results, building on existing work and having role delineation.

**Table 1:** Summary of recommendations: Consultation for Establishment of NPS [12]

Delineate its role from those of APAC, PHARM and others and that chairs of these groups be invited to attend NPS Board meetings as a means of sharing information	
Provide national leadership in promoting consistency of information in independent medicines information products	
Coordinate the development of a National Medicines Information Phone-Line Network for consumers and health professionals	
Consult with medical educators to encourage an increased focus on quality prescribing	
Coordinate a National Academic Detailing Program, linking with Divisions of General Practice	
Provide national leadership to increase the level of computerised prescribing by GPs and other medical practitioners	
Provide leadership in developing incentives for high quality prescribing	
Explore and develop means for providing locally relevant prescriber feedback	
Auspice a program of research of initiatives to improve continuity of care across sectors and between professions	
Explore models to improve communication between GPs and pharmacists	
Coordinate a national communications and community awareness strategy to support QUM for consumers	
Develop constructive partnerships with pharmaceutical industry to further safe and appropriate use of medicines	
Find options for making services available to consumers and for supporting consumer based activities.	

The company started with 26 member organisations in March 1998 (Table 2). The Minister noted that NPS would be independent, collaborative and driven from the bottom-up [13].

**Table 2:** Founding Member Organisations of National Prescribing Service

Australian & New Zealand College of Anaesthetists	Australian Pensioner & Superannuants Federation
Australasian Society of Clinical & Experimental Pharmacologists and Toxicologists	Australian College of Dermatologists
Australian Council of Social Services	Australian General Practice Network
Australian Healthcare and Hospitals Association	Australian Medical Association
Medicines Australia	Australian Private Hospitals Association
Carers Australia	Consumers Health Forum of Australia
Council on the Ageing	Commonwealth Department of Health and Aged Care
Commonwealth Department of Veterans Affairs	Health Consumers of Rural and Remote Australia
National Aboriginal Community Controlled Health Organisation	Pharmaceutical Society of Australia
Pharmacy Guild of Australia	Australian Self-Medication Industry
Royal Australian and New Zealand College of Psychiatrists	Royal Australian College of General Practitioners
Royal Australian College of Physicians	Australian College of Nursing
Rural Doctors Association of Australia	Society of Hospital Pharmacists of Australia

### Governance

The original board of directors was drawn from five categories of members: government, general practice, specialist prescribers, consumers and pharmacy. Over time the board became skills-based while retaining perspectives consistent with NPS’s membership. Directors were nominated by member organisations and selected by the Board through a robust application and interview process.

The not-for-profit structure with funding from government, responsible for its own strategy and accountable to its membership has served the company well. NPS is well connected to health policy, has flexibility to act autonomously and has built credibility with health professionals and the community.

### Evolving scope of activity

#### Evidence based interventions

NPS introduced its first program, management of peptic ulcer and eradication of *H.pylori* in late 1998 with: *NPS News*; feedback reports to general practitioners (GPs) comparing their prescribing with peers; and a case study.

In 1999, a national academic detailing program began with a small group trained to visit GPs on two topics: management of respiratory infection and chronic obstructive pulmonary disease. Since that time over 500 academic detailers have been trained and they have delivered over 300,000 visits for GPs on many topics.

While NPS has been committed to academic detailing as an intervention because of its strong evidence base [14], there was demand for facilitated, interactive case-based meetings and these were added as a service offering.

Another early intervention was clinical audit: self-audit tools that supported GPs to review management of a cohort of patients, compare themselves to colleagues and guidelines and reflect on the changes for individual patients. NPS has conducted 51 clinical audits, for 61,673 GPs with the most popular topics being antibiotics, hypertension and diabetes.

NPS worked with ASCEPT to create a national prescribing curriculum to teach medical students prescribing. This was later adapted for nursing and pharmacy students and underpinned with a *National Prescribing Competency Framework* [15].

Publications introduced new evidence to health professionals. *Australian Prescriber* [16] and *RADAR* [17], a summary of the ‘place in therapy’ for new medicines listed on the Pharmaceutical Benefits Scheme [PBS], were both important additions to NPS.

#### Consumer programs

Consumer programs were specifically funded from 2003 to raise community awareness of QUM principles. This work included: antibiotic awareness [18]; *Be MedicineWise Week*; *MedicineList* on a card and in a smart phone application; and collaborations with grass roots organisations. A peer-education program for seniors run by the Council on the Ageing [COTA] was found to improve people’s confidence in how they used medicines [19]. Other programs were provided for people from culturally and linguistically diverse communities, using peer-educators, local health professionals and through ‘in language’ community radio.

The *Good Medicines Better Health* program, developed with the National Aboriginal Community Controlled Health Organisation [NACCHO] and co-designed by Aboriginal Health Workers produced QUM modules that were used in 50 Aboriginal Medical Services. Evaluation found that Aboriginal Health Workers demonstrated improved knowledge and skill immediately following and many months after the training. They spoke of improved confidence in discussing medicines, especially with doctors [20].

A phone service for the general public delivered by pharmacists, called *Medicines Line*, provided information for consumers with appropriate referral to health professionals. The service answers some 8000 calls annually and has a strong social media presence.

#### Medical tests

In 2009, NPS extended activity to include quality referrals for diagnostic tests. This built on relationships in general practice and deployment of evidence-based interventions. Audit, feedback and academic detailing have been used to address: imaging for low back pain; vitamin D testing; vitamin B12/folate deficiency; and imaging for ankle and knee pain.

Recently NPS has facilitated *Choosing Wisely Australia*™ which engages medical and health colleges to reduce low value care [21].

#### Big data

In May 2011, NPS received funding for a new service to monitor use of medicines in general practice [22, 23]. *MedicineInsight* includes 650 general practices, involving 3300 GPs and 3.6 million regular patients. The two primary uses of the data are quality improvement in participating practices and post-market surveillance of medicines and tests. The data are used to inform primary care policy, monitor new medicines and vaccines, evaluate new models of care and inform regulatory and subsidy decisions.

### Measuring impact

Program evaluation has been a priority, including: formative research and program logic; process evaluation; impacts on knowledge, attitudes and behaviours; and outcome evaluations [24, 25].

NPS’s contracts with government have required annual savings on Pharmaceutical Benefits Scheme (PBS) and Medical Benefits Schedule (MBS) expenditure, resulting in a total of $998.74 million in PBS savings since 1998 and $97.88 million in MBS savings since 2010.

Savings are calculated using time series methods, provided:

- program objectives would result in reductions in drug or test ordering
- impact can be detected using established time series methods [26, 27]
- sufficient data are available (typically 12 months from program launch)
- annual expenditure is substantial [28].

**Fig. 1:**
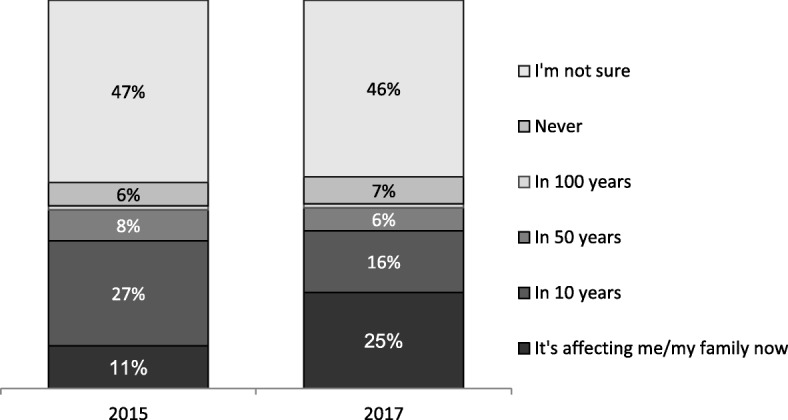
Knowledge of when antibiotic resistance will affect them and their families based on annual surveys of the general public (*n* = 2500) [31]

**Fig. 2:**
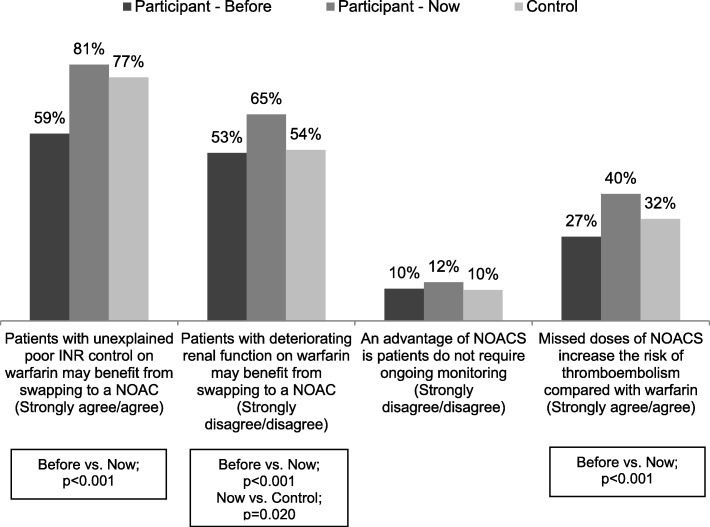
Correct response to knowledge statements about warfarin and new oral anticoagulants (NOACS) by program participants before and after the 2013 program compared with control GPs [32].

**Fig. 3:**
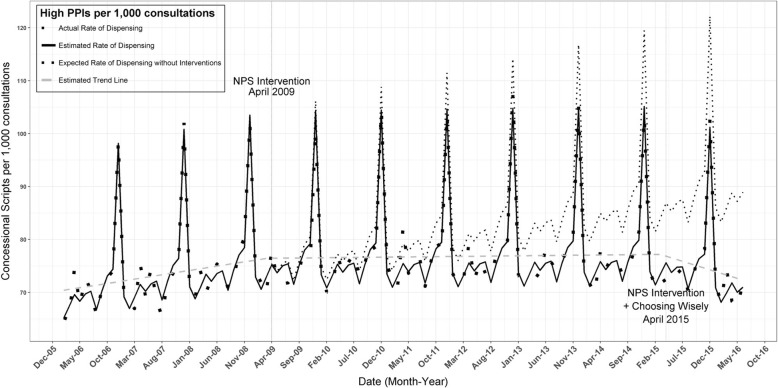
Rate of high strength proton pump inhibitors (PPI) dispensed per 1,000 consultations, December 2005 to October 2016 [31]. Following the 2009 NPS MedicineWise program there was a 6.7% reduction in the dispensing rate of high strength PPIs by March 2015 and an 8.6% reduction by June 2016 [31]. Choosing Wisely released a PPI recommendations for GPs in 2015. Source PBS

**Fig. 4:**
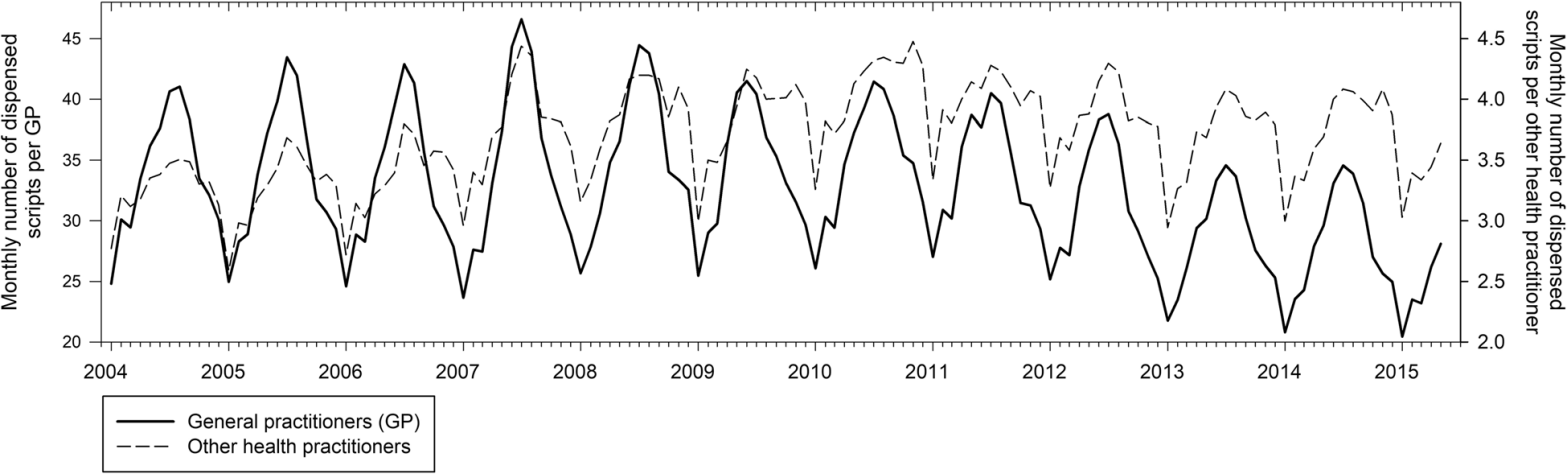
Reductions in antibiotic prescribing in general practice compared with other health professionals associated with annual winter programs to improve management of respiratory tract infections [33]

**Fig. 5:**
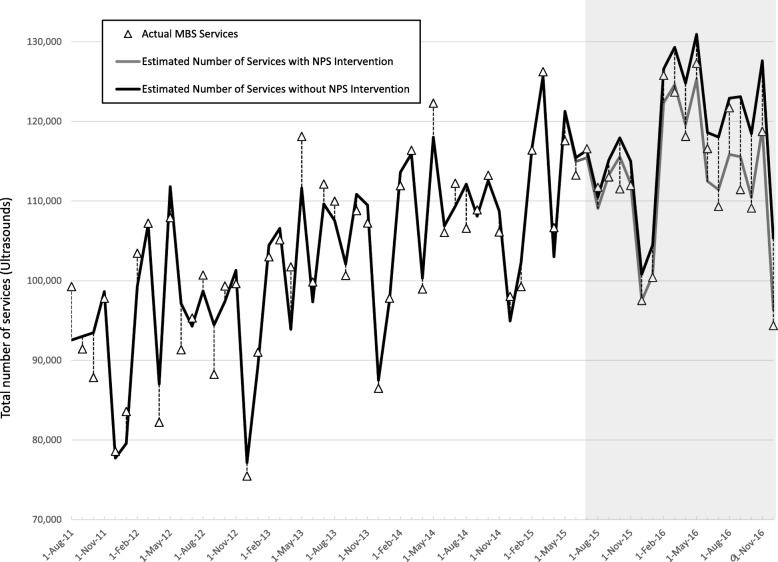
Time series analysis of monthly count of ultrasound of the abdomen service, 1 August 2011 to 31 December 2016 showing impact of NPS intervention launched mid-2015 [29]

Other important impacts have included changes in community attitudes to antimicrobial resistance (Fig. 1); changes in knowledge and attitudes of GPs (Fig. 2); and shifts in prescribing (Figs. 3 and 4) and referrals for medical tests (Fig. 5).

#### Health and economic outcomes

**Fig. 6:**
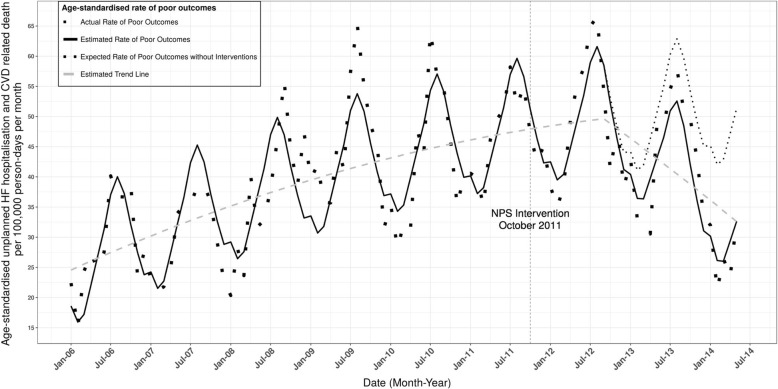
Unplanned hospitalisations for heart failure and deaths due to cardiovascular diseases per 100,000 person-days per month from January 2006 to June 2014, 45 and Up Study participants [29]. The study showed statistically significant change in the monthly number of participants per 100,000 person-days dispensed HF specific beta-blockers and aldosterone antagonist in line with the program messages. There was also a large reduction in the rate of unplanned hospitalisations and CVD related deaths following a delay of 12 months after the intervention date. Data source: 45 Up Study, Sax Institute, Australia [29].

A health outcome study found a reduction in poor patient outcomes, defined as unplanned hospitalisation and death due to cardiovascular disease, in patients with heart failure who had improved medicines management as a result of a 2011 program that increased use of ACE inhibitors/angiotensin II inhibitors and beta blockers [29] (Fig. 6). Other work has modelled reductions in complications of diabetes, shown fewer strokes from atrial fibrillation [30] and economic advantages of improving asthma management [29].

### Looking to the future

In 2018, NPS has 46 members, 300 staff, provides services to over 24,000 GPs and 8000 pharmacists every year, interacts with over a million citizens and saves government expenditure. It also has a subsidiary, *VentureWise* to access commercial customers and ensure financial stability.

The coming years will bring new opportunities in high cost medicines and precision medicine, engagement with specialists, better use of data and support for new models of care.

## Conclusions

Features of NPS’s success have included taking a service-based rather than authoritarian approach to general practice to build trust and credibility. Evaluation has helped the organisation learn and has ensured renewal of 3–4 yearly contracts with government. Partnerships have added value and reduced duplication allowing for more delivery on mission.

There remain many opportunities and by remaining grounded in its purpose and health consumer roots, NPS will continue to add value.
